# Correction: An observational study comparing HPV prevalence and type distribution between HPV-vaccinated and -unvaccinated girls after introduction of school-based HPV vaccination in Norway

**DOI:** 10.1371/journal.pone.0226706

**Published:** 2019-12-12

**Authors:** The PLOS ONE Staff

The following information is missing from the Competing Interests statement: WHO-IARC: Where authors are identified as personnel of the International Agency for Research on Cancer / World Health Organization, the authors alone are responsible for the views expressed in this article and they do not necessarily represent the decisions, policy or views of the International Agency for Research on Cancer / World Health Organization.

Norwegian Immunization Registry SYSVAK (Norwegian Institute of Public Health) provided data, but is not responsible for the analyses and interpretation in this article.

The publisher apologizes for the error.
