# Supplementary figures and images for: An observational study comparing HPV prevalence and type distribution between HPV-vaccinated and -unvaccinated girls after introduction of school-based HPV vaccination in Norway
Source: PLoS One. 2019 Oct 10;14(10):e0223612. doi: 10.1371/journal.pone.0223612 (PMC6786612; doi:10.1371/journal.pone.0223612)

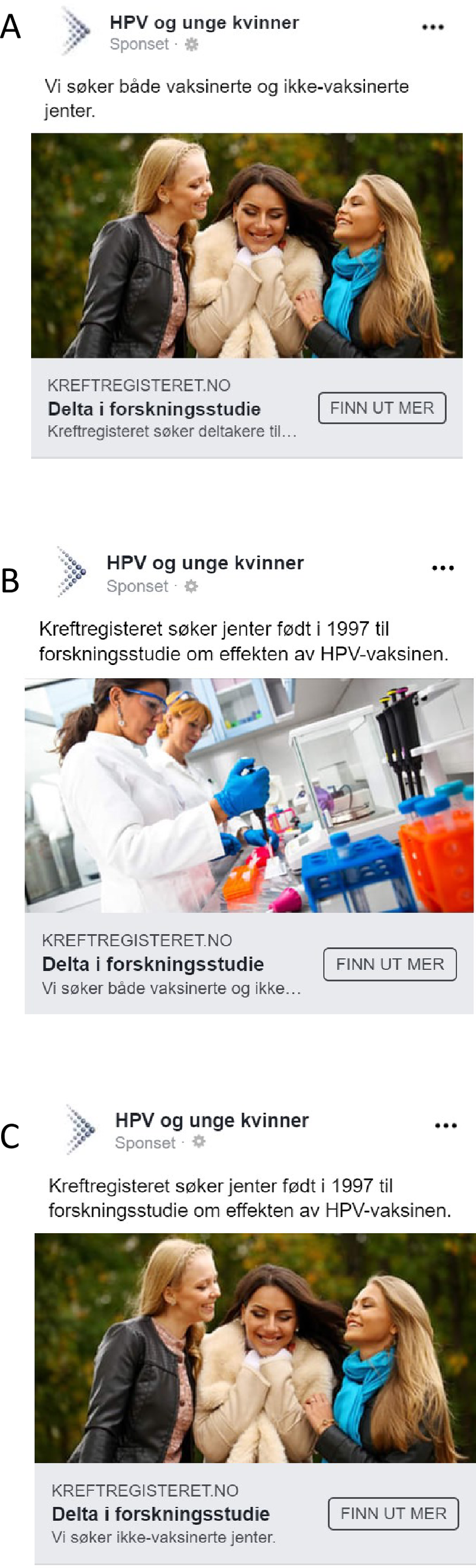

Supplement: S1 Fig — (A-B) Examples of advertisements used to target both vaccinated and unvaccinated. (C) Advertisement used to target only unvaccinated girls. Credits for images: Shutterstock.com (TIF) [file pone.0223612.s001.tif]
