# Supplementary material for: An observational study comparing HPV prevalence and type distribution between HPV-vaccinated and -unvaccinated girls after introduction of school-based HPV vaccination in Norway
Source: PLoS One. 2019 Oct 10;14(10):e0223612. doi: 10.1371/journal.pone.0223612 (PMC6786612; doi:10.1371/journal.pone.0223612)
Supplement: S1 Table — A participant is defined as vaccinated if she received at least one dose of quadrivalent HPV vaccine. (DOCX) [file pone.0223612.s002.docx]

S1 Table. Type-specific vaginal human papillomavirus (HPV) prevalence by HPV vaccination status. A participant is defined as vaccinated if she received at least one dose of quadrivalent HPV vaccine.

|  | Prevalence (95% CI) | | | | | |  |  |
| --- | --- | --- | --- | --- | --- | --- | --- | --- |
|  | Vaccinated | | | Unvaccinated | | | Prevalence ratio (95% CI) | Adjusted prevalence ratio (95% CI) |
|  | N | % | 95% CI | N | % | 95% CI |  |  |
| **Total** | 246 | 100 |  | 68 | 100 |  |  |  |
| Any HPV type | 97 | 39.4 | (33.3-45.8) | 26 | 38.2 | (26.7-50.8) | 1.03 (0.67-1.59) | 0.96 (0.62-1.48) |
| HPV16 or 18 | 2 | 0.8 | (0.1-2.9) | 2 | 2.9 | (0.3-10.2) | 0.28 (0.04-1.96) | 0.24 (0.03-1.84) |
| HPV6,11,16 or 18 | 2 | 0.8 | (0.1-2.9) | 4 | 5.9 | (1.6-14.4) | 0.14 (0.03-0.75) | 0.10 (0.02-0.60) |
| High-risk types | 48 | 19.5 | (14.8-25.0) | 13 | 19.1 | (10.6-30.5) | 1.02 (0.55-1.88) | 0.96 (0.52-1.79) |
| Low-risk types | 81 | 32.9 | (27.1-39-2) | 20 | 29.4 | (19.0-41.7) | 1.12 (0.69-1.83) | 1.03 (0.63-1.68) |
| Non-vaccine-targeted types | 96 | 39.0 | (32.9-45.4) | 25 | 36.8 | (25.4-49.3) | 1.06 (0.68-1.65) | 0.99 (0.63-1.54) |

Participants with multiple infections were counted in each category in which their type-specific HPV infection(s) belonged. CI: confidence interval
