# Supplementary material for: An observational study comparing HPV prevalence and type distribution between HPV-vaccinated and -unvaccinated girls after introduction of school-based HPV vaccination in Norway
Source: PLoS One. 2019 Oct 10;14(10):e0223612. doi: 10.1371/journal.pone.0223612 (PMC6786612; doi:10.1371/journal.pone.0223612)
Supplement: S2 Table — A participant is defined as vaccinated if she received at least one dose of the bivalent or the quadrivalent HPV vaccine at least the calendar year before sexual debut. (DOCX) [file pone.0223612.s003.docx]

S2 Table. Type-specific vaginal human papillomavirus (HPV) prevalence by HPV vaccination status. A participant is defined as vaccinated if she received at least one dose of the bivalent or the quadrivalent HPV vaccine at least the calendar year before sexual debut.

|  | Prevalence (95% CI) | | | | | |  |  |
| --- | --- | --- | --- | --- | --- | --- | --- | --- |
|  | Vaccinated | | | Unvaccinated | | | Prevalence ratio (95% CI) | Adjusted prevalence ratio (95% CI) |
|  | N | % | 95% CI | N | % | 95% CI |  |  |
| **Total** | 240 | 100 |  | 72 | 100 |  |  |  |
| Any HPV type | 92 | 38.3 | (32.2-44.8) | 30 | 41.7 | (30.2-53.9) | 0.92 (0.61-1.39) | 0.97 (0.63-1.48) |
| HPV 16 or 18 | 1 | 0.4 | (0.0-2.3) | 3 | 4.2 | (0.9-11.7) | 0.10 (0.01-0.96) | 0.11 (0.01-1.30) |
| HPV 6.11.16 or 18 | 1 | 0.4 | (0.0-2.3) | 5 | 6.9 | (2.3-15.5) | 0.06 (0.01-0.51) | 0.05 (0.00-0.42) |
| High risk types | 46 | 19.2 | (14.4-24.7) | 14 | 19.4 | (11.1-30.5) | 0.99 (0.54-1.79) | 1.07 (0.58-1.99) |
| Low risk types | 77 | 32.1 | (26.2-38.4) | 23 | 31.9 | (21.4-44.0) | 1.00 (0.63-1.60) | 1.03 (0.64-1.67) |
| Non-vaccine types | 92 | 38.3 | (32.2-44.8) | 28 | 38.9 | (27.6-51.1) | 0.99 (0.65-1.50) | 1.04 (0.67-1.61) |

Participants with multiple infections were counted in each category in which their type-specific HPV infection(s) belonged. CI: confidence interval
