# Supplementary material for: An observational study comparing HPV prevalence and type distribution between HPV-vaccinated and -unvaccinated girls after introduction of school-based HPV vaccination in Norway
Source: PLoS One. 2019 Oct 10;14(10):e0223612. doi: 10.1371/journal.pone.0223612 (PMC6786612; doi:10.1371/journal.pone.0223612)
Supplement: S3 Table — A participant is defined as vaccinated if she received at least three doses the quadrivalent HPV vaccine at least the calendar year before sexual debut. (DOCX) [file pone.0223612.s004.docx]

S3 Table. Type-specific vaginal human papillomavirus (HPV) prevalence by HPV vaccination status. A participant is defined as vaccinated if she received at least three doses the quadrivalent HPV vaccine at least the calendar year before sexual debut.

|  | Prevalence (95% CI) | | | | | |  |  |
| --- | --- | --- | --- | --- | --- | --- | --- | --- |
|  | Vaccinated | | | Unvaccinated | | | Prevalence ratio (95% CI) | Adjusted prevalence ratio (95% CI) |
|  | N | % | 95% CI | N | % | 95% CI |  |  |
|  | 220 | 100 |  | 92 | 100 |  |  |  |
| Any HPV type | 79 | 35.9 | (29.6-42.6) | 43 | 46.7 | (36.3-57.4) | 0.77 (0.53-1.11) | 0.83 (0.55-1.24) |
| HPV 16 or 18 | 1 | 0.5 | (0.0-2.5) | 3 | 3.3 | (0.68-9.2) | 0.14 (0.01-1.34) | 0.23 (0.02-2.68) |
| HPV 6,11,16 or 18 | 1 | 0.5 | (0.0-2.5) | 5 | 5.4 | (1.8-12.2) | 0.08 (0.01-0.72) | 0.08 (0.01-0.75) |
| High risk types | 39 | 17.7 | (12.9-23.4) | 21 | 22.8 | (14.7-32.8) | 0.78 (0.46-1.32) | 0.86 (0.49-1.54) |
| Low risk types | 67 | 30.5 | (24.4-37.0) | 33 | 35.9 | (26.1-46.5) | 0.85 (0.56-1.29) | 0.89 (0.57-1.40) |
| Non-vaccine types | 79 | 35.9 | (29.6-42.6) | 41 | 44.6 | (34.2-55.3) | 0.81 (0.55-1.18) | 0.87 (0.58-1.30) |

Participants with multiple infections were counted in each category in which their type-specific HPV infection(s) belonged. CI: confidence interval
